# Spelling Acquisition in English and Italian: A Cross-Linguistic Study

**DOI:** 10.3389/fpsyg.2015.01843

**Published:** 2015-12-08

**Authors:** Chiara V. Marinelli, Cristina Romani, Cristina Burani, Pierluigi Zoccolotti

**Affiliations:** ^1^Department of Psychology, University of Rome La SapienzaRome, Italy; ^2^Neuropsychological Research Centre, IRCCS Fondazione Santa LuciaRome, Italy; ^3^School of Life and Health Science, Aston UniversityBirmingham, UK; ^4^ISTC Institute for Cognitive Sciences and Technologies, Consiglio Nazionale delle RicercheRome, Italy; ^5^Department of Life Sciences, University of TriesteTrieste, Italy

**Keywords:** spelling acquisition, orthographic consistency, cross-linguistic comparison, lexical effects, children

## Abstract

We examined the spelling acquisition in children up to late primary school of a consistent orthography (Italian) and an inconsistent orthography (English). The effects of frequency, lexicality, length, and regularity in modulating spelling performance of the two groups were examined. English and Italian children were matched for both chronological age and number of years of schooling. Two-hundred and seven Italian children and 79 English children took part in the study. We found greater accuracy in spelling in Italian than English children: Italian children were very accurate after only 2 years of schooling, while in English children the spelling performance was still poor after 5 years of schooling. Cross-linguistic differences in spelling accuracy proved to be more persistent than the corresponding ones in reading accuracy. Orthographic consistency produced not only quantitative, but also qualitative differences, with larger frequency and regularity effects in English than in Italian children.

## Introduction

The dual route models (e.g., Ellis, 1984; Barry and Seymour, 1988; Kreiner, 1992) assume the existence of two spelling procedures: the lexical procedure, in which word-specific orthographic representations are accessed, and the sub-lexical procedure, which relies on the serial conversion of phonemes into graphemes according to specific rules. In a developmental perspective, it is generally held that beginners firstly rely on the non-lexical phoneme-to-grapheme conversion procedure and only subsequently shift to the lexical one (e.g., Frith, 1985). Indeed, phonological decoding would be instrumental in acquiring the orthographic representation of words (Perfetti, 1992; Share, 1995, 1999; Ehri, 1998; Sprenger-Charolles et al., 1998; Shahar-Yames and Share, 2008), and then, in building up the orthographic lexicon. However, if children know the words to be spelled, lexical spelling is also used among young children (e.g., Cossu et al., 1995; Frith et al., 1998; Angelelli et al., 2010; Notarnicola et al., 2011). Despite this early evidence of lexical spelling, it is generally held that reliance on this procedure increases as children become more competent and acquire a relatively great amount of orthographic representations (e.g., Sprenger-Charolles et al., 2003).

Orthographic consistency may influence reliance on the lexical or sub-lexical procedures in literacy acquisition as a function of age. Cross-linguistic differences have been examined in greater depth with regard to reading: orthographic consistency is a crucial factor for determining the rate and the modality of reading acquisition across different languages (for a review, see Ziegler and Goswami, 2005). Inconsistent orthography-phonology mappings in opaque orthographies are associated to a longer time to acquire reading (e.g., Seymour et al., 2003) and greater reliance on lexical procedure/larger units of conversion compared to consistent orthographies (e.g., Ziegler et al., 2001). The orthographic regularity of the language might play an important role also in spelling acquisition.

There may be different reasons to suppose that orthographic consistency modulates spelling acquisition to a greater extent than reading acquisition. Compared to reading, spelling requires greater processing demands (Bosman and Van Orden, 1997), as well as greater reliance on orthographic knowledge; i.e., it is more sensitive to deficits in orthographic knowledge (Curtin et al., 2001). In this vein, cross-linguistic differences in the mastering of spelling may be expected to be partially different from those described for reading. However, spelling acquisition has received much less attention than reading acquisition and cross-linguistic studies examining the role of orthographic consistency on spelling have been quite limited.

In fact, the literature on spelling acquisition and impairment is prevalently “Anglo-centric” (Wimmer and Landerl, 1991; Caravolas, 2004; Share, 2008). The few studies that have investigated cross-language differences with respect to the properties of the orthography in spelling different types of stimuli (Wimmer and Landerl, 1991; Caravolas and Bruck, 1993; Bruck et al., 1996, unpublished study quoted by Caravolas, 2004), indicated in general faster acquisition in spellers of regular orthographies than in spellers of English (e.g., Caravolas and Bruck, 1993). Thus, children learning Czech (a shallow orthography) were more accurate than English children in non-word spelling after 8 months of schooling, even if they began first grade with less preschool exposure to pre-literacy skills than English children (Caravolas and Bruck, 1993). Then, the consistency in phoneme-grapheme mappings makes the acquisition of the non-lexical procedure faster in regular than in irregular orthographies (Caravolas and Bruck, 1993). Similar findings come from a study examining the ability to spell simple, doubled and cluster consonants in 3rd and 4th grade Danish and Icelandic children (Juul and Sigurdsson, 2005). The two languages have similar orthographic structure but Danish is more inconsistent than Icelandic. The task consisted in adding the missing letter(s) over the underscore in order to complete the non-words dictated to the children (e.g., ja__e (jamme) /'jɑmə/). Cross-linguistic differences did not emerge in inserting simple onset consonants, in which children of both languages were generally close to ceiling. However, better performance in inserting doubled and cluster consonants was found among Icelandic than Danish (particularly younger) children. Notably, Icelandic 3rd graders outperformed Danish 4th graders even if the latter were 2 years older, and had a performance similar to 6th grade Danish children. Juul and Sigurdsson (2005) concluded that orthographic inconsistency might be considered as a kind of handicap for children learning to spell, producing a delayed acquisition of the sub-lexical procedure. Wimmer and Landerl (1991) examined 1st grade German and English speaking children in a task requesting to insert the missing ambiguous vowels (i.e., not predictable on the basis of phonology but only of lexical knowledge) into the skeleton spelling of words. Words had the same meaning and similar phonemic and orthographic structures in the two languages (e.g., boat-boot, rose-rose). English speaking children produced more errors and a larger variety of incorrect alternatives than German speaking children indicating that orthographic inconsistency in the mappings (and the higher number of alternatives homophonic to the correct one) made the choice of the correct alternative more difficult. Then, the greater difficulty of inconsistent orthographies makes spelling acquisition particularly difficult in the early phases of literacy acquisition. Few studies examined how spelling performance develops with age as a function of the orthography and/or whether reliance on the two spelling procedures changes with age, an issue investigated in the present study.

Orthographic consistency might influence not only the ease of spelling acquisition but also the reliance on different spelling procedures at later stages of spelling development (Caravolas, 2004). Inconsistent phoneme-grapheme mappings in opaque orthographies might induce children to rely more on the lexical procedure in order to produce accurate spellings (impossible to obtain through non-lexical conversion). By contrast, in shallow orthographies children might obtain reasonable levels of accuracy also using the non-lexical procedure. Notably, previous cross-linguistic studies focused on a single task or stimulus material (e.g., non-words) and investigations examining a wide spectrum of psycholinguistic variables (such as frequency, lexicality, length, etc.) are lacking. Such information might be important in characterizing the reliance on lexical vs. non-lexical procedures in spelling as a function of age in languages with different levels of orthographic consistency.

The present study is part of a larger investigation devoted to understanding mechanisms and cognitive correlates of literacy acquisition in Italian (a language with a mostly regular orthography) and English (a language with high orthographic irregularity). In the present report, we focus on spelling acquisition as a function of orthography consistency, but we also refer to similarities/differences with the acquisition of reading in the two languages. Note that Italian is extremely consistent in reading, while in spelling there are a number of inconsistent words (for example [kwore], “heart,” may be spelt in two phonologically plausible ways, such as *cuore* and *quore*, but only the first solution is also orthographically correct; see Luzzatti et al., 1994, for further examples). The presence of this relatively small set of ambiguous words in spelling allows examining the reliance on lexical spelling also in a shallow orthography such as Italian, in order to compare it with the reliance on the lexical procedure in English.

The limited evidence on Italian suggests greater reliance on non-lexical than lexical processing among Italian children as well as longer time to acquire lexical spelling compared to the sub-lexical one. In a study from second to eighth grade, Tressoldi (1996) reported more errors in spelling pseudo-homophones than non-words across all grades, indicating greater reliance on non-lexical than lexical processing among Italian children. Notarnicola et al. (2011) examined spelling acquisition from 1st to 8th grade Italian children and found that although Italian children used lexical, as well non-lexical, spelling from the first year of school, relative reliance on these procedures was optimized at different ages. Children were quite accurate (about 90%) on regular words already by 1st grade; by contrast, accuracy in spelling irregular words reached levels of performance similar to the other types of stimuli only by 6th–7th grade. These data indicate an earlier and more rapid development for the sub-lexical procedure and a more gradual acquisition of the lexical procedure, consistently with other regular orthographies, such as Czech, Turkish, German, and Spanish (for a review, see Caravolas, 2004). The analyses on the error types generally confirmed these differential trends: phonologically plausible errors were prevalent at all grades, while all other types of errors decreased with schooling, being present only in first to third grade.

In evaluating the acquisition of spelling skills in two quite different languages, such as English and Italian, it is fundamental to have orthographic materials that are comparable between the two languages. For this reason, in the present study lists of words and non-words were generated matching the two languages for as many psycholinguistic variables as possible. In order to evaluate both lexical and non-lexical processing the effects of regularity, frequency, lexicality, and length were examined. According to the Dual Route Cascaded Model (DRC, Coltheart et al., 2001; for spelling: e.g., Barry, 1994), the effects of lexicality (words spelled better than non-words) and frequency (high spelled better than low frequency words) may be considered as markers of the lexical procedure with a greater reliance on this procedure for stimuli for which it is easier to acquire the specific orthographic representations, as in the case of words and especially high frequency words, compared to non-words and low frequency words. On the other hand, the regularity (regular spelled better than irregular words) and length (short spelled better than long words) effects may be considered as markers of the sub-lexical procedure. In fact, the regularity effect should be greater if children use the non-lexical procedure; this would allow a good accuracy in spelling regular words while producing a large number of phonological plausible errors (i.e., stimuli homophonic to the correct spelling but orthographically incorrect) in spelling irregular words. The length effect reflects more the serial process of phoneme-to-grapheme conversion (with lower accuracy when the number of letters to be converted increases) than the lexical spelling^1^.

Several questions emerge from these observations: does orthographic consistency modulate the characteristics of literacy and the reliance on the lexical or sublexical procedure in spelling? Are crosslinguistic differences long-lasting or are on the foreground only in the initial phases of spelling acquisition? In the present study, we aimed at studying spelling acquisition among English and Italian children during elementary school. We focused on the spelling ability after some (at least 2 years) training had been received by the child and also later in the course of elementary school. Due to the different times of starting formal instruction in Italy and England (when children are, on average, 6 and 5 years old, respectively), we matched children both on the basis of chronological age and in terms of years of schooling. Same age “younger” children attended third grade in England and second grade in Italy; same age “older” children attended fifth grade in England and fourth grade in Italy. The inclusion in the study of Italian children attending fifth grade allowed disentangling the influence of school attendance from that of chronological age. In general, we expected that, due to the inconsistency of their orthography, English children would present more spelling errors than Italian children. As to the profile of responding, we expected that younger English children would resemble Italian children; with greater exposition to the orthographic inconsistency and spelling (and reading) experience, we expected a larger use of the lexical procedure among English children (and then larger frequency and lexicality effects) than Italian children. In particular, we hypothesize that, by prolonged exposure to an inconsistent orthography, older English children learn to place a greater reliance on the lexical procedure that may allow higher levels of accuracy with respect to the grapheme-to-phoneme conversion procedure.

Additionally, data on spelling acquisition will be compared to reading data in order to examine whether orthographic consistency modulates in different ways reading and spelling. This comparison was possible since in the reading part of the present study we used the same set of regular stimuli (assessing the frequency, lexicality, and length effects) used for the assessment of spelling. By contrast, note that the regularity effect was examined only in spelling, due to the absence of irregular words in Italian reading. To the extent in which spelling requires greater processing demands than reading (Bosman and Van Orden, 1997), particularly in the case of lexical representations (Tainturier and Rapp, 2001), we expected a larger disadvantage in spelling than reading, even after several years of formal literacy instruction, an effect more evident among English children compared to Italian children.

## Method

### Participants

Criteria for inclusion in the English and Italian sample were: absence of neuro-sensory deficits or cognitive impairment (according to Raven's Colored Progressive Matrices)^2^, adequate socio-educational conditions and opportunities of literacy acquisition. Children were randomly selected from local public primary schools of the Birmingham area (for the English sample) and Rome and Naples areas (for the Italian sample).

As it regards the chronological match, younger children attended 3rd grade in England and 2nd grade in Italy; older children attended 5th grade in England and 4th grade in Italy. One hundred and seventy-seven Italian (87 F, 90 M) and 79 English children (41 F, 38 M) participated to the study. Younger children were 90 in Italian (43 F, 47 M, mean age = 7.3 year) and 39 in England (16 F, 23 M, mean age = 7.8 years); older children were 87 in Italy (44 F, 43 M, mean age = 9.6 years) and 40 in England (25 F, 15 M, mean age = 9.9 year). Matched groups did not differ for gender (χ^2^ < 1), but were different for age [for younger children: *t*_(128)_ = 12.73, *p* < 0.0001; for older children: *t*_(126)_ = 7.73, *p* < 0.0001] with English children older by ca. 4 months^3^. Raven performance was slightly higher among English children (for younger children: *t*_(128)_ = 4.12, *p* < 0.0001; for older children: *t*_(126)_ = 3.45, *p* < 0.001].

Additionally, a group of 30 Italian children (17 F, 13 M) attending 5th grade (mean age = 10.6 years) were also examined in order to match the years of schooling of the 5th grade English children. When English and Italian groups were compared on the basis of years of schooling (5th grade), they did not differ for gender (χ^2^ < 1) and Raven performance (*t* < 01) but, as for the younger groups, they differed for age [*t*_(69)_ = 9.19, *p* < 0.0001], with the Italian children older by ca. 6 months.

Parents were informed about the experimental procedure and authorized the participation of their son/daughter to the study. The study was conducted according to the principles of the Helsinki Declaration and was approved by the local committee of the Departments and by the school authorities.

### Materials

Two lists of stimuli for each language were prepared. The first list comprised 120 stimuli and assessed the effects of length (4, 5, 6, and 7–9 letter items) and type of stimuli (high frequency words, low frequency words and non-words) for a total of 10 stimuli in each sub-set (see Appendix 1 in Supplementary Material). High frequency words had a mean frequency of 59.4 (*SD* = 88.8) in English (CELEX lexical database, Baayen et al., 1993) and 63.7 (*SD* = 55.6) in Italian (Colfis database^4^, Bertinetto et al., 2005); low frequency words had a mean frequency of 2.9 (*SD* = 1.4) in English and of 3.2 (*SD* = 3.2) in Italian. Words were all nouns with regular correspondence between phonemes and graphemes. There were only Italian words with a regular stress (i.e., on the penultimate syllable)^5^ and English words with regular correspondences according to Hanna et al. (1966). The sets were balanced for the presence of double consonants, clusters of consonants and of contextual rules (Barca et al., 2007). Non-words were created from high frequency words changing one to three letters. Non-words had the same ortho-syllabic difficulty of words (in terms of presence of double consonants, clusters of consonants and of contextual rules).

The second list assessed the effect of regularity, and included 30 regular words and 30 irregular words (see Appendix 2 in Supplementary Material). In English, words were considered irregular if they had an infrequent phoneme-grapheme correspondence (with a frequency of less than 5% according to Hanna et al., 1966); in Italian, irregular words were words with the graphemes QU/CU/CQU or SCE/SCIE-CE/CIE, GE/GIE that may be spelt in different ways based on phonology and require lexical procedure to be spelt correctly (Luzzatti et al., 1994). Words were of high frequency (in Italian: mean = 74, *SD* = 140 according to Colfis; Bertinetto et al., 2005; in English: mean = 64.6, *SD* = 81 according to CELEX lexical database; Baayen et al., 1993). On average, words were 6.8 letter long (*SD* = 0.9 for English and 7.6 for Italian stimuli, respectively). Irregular words were matched with regular words for ortho-syllabic difficulty (presence of double consonants, clusters of consonants and contextual rules), number of letters and word frequency.

### Procedure

Words were presented for dictation individually to the children. Each word was read in a clear, loud and neutral voice, without emphasizing the source of the spelling difficulty. Children had to repeat the words in order to assess they had heard the stimulus correctly. In the rare cases in which children repeated the stimulus incorrectly (presumably because they did not perceive it correctly), the word/non-word was dictated again after about 20–30 stimuli.

Words and non-words were presented in separate blocks. To avoid priming of non-words from the words they were derived from, the non-word block was presented before the word blocks. Non-words were dictated in a single quasi-random order. Stimuli from the two sets of words were randomized and presented intermixed together in a single quasi-random order. To make the task not too tiring, words were divided into two separate blocks with a brief pause between them.

On a separate day, children were administered a reading test comprising the same sets of words and non-words of the first list described above. Children were presented stimuli (either words or non-words) singly on the center of a PC screen and were asked to read them aloud as fast and as accurately as possible. Both RTs and errors were recorded; only accuracy measures will be considered in this report (for more details on the procedure see Marinelli et al., submitted). At least 20 days elapsed between the reading and spelling tests. In about half of cases, children performed the reading test first and the spelling test afterwards; in the other half, the opposite order was followed. No detectable differences were found between the two groups with different task orders (Fs not significant).

### Data analysis

Spelling data (% of errors) were submitted to three separate ANOVAs. The first ANOVA included word frequency (high, low) and length (4, 5, 6, 7–9 letters) as repeated measures; the second one lexicality (words, non-words) and length (4, 5, 6, 7–9 letters) as repeated measures; and the third one regularity (regular, irregular words). In each analysis, age (young and old children) and language (English and Italian) were entered as between group variables. The same three analyses were also performed for comparing children on the basis of the number of years of schooling (5th grade Italian and 5th grade English children). In this case language (English and Italian) was the only between group variable. In both age and years of schooling comparisons, interactions were explored with planned comparisons.

Size effects were computed as partial eta squared (η^2^); Cohen (1988, p. 283) indicates 0.0099 as a reference point for a small effect, 0.0588 for a medium effect and 0.1379 for a large effect. For the sake of comparison, we present the size effects for similar analyses carried out on reading accuracy for the frequency and lexicality analyses on the same stimuli (Marinelli et al., submitted). Such comparison was not possible in the case of the regularity effect since different forms of irregularity were examined in the case of reading and spelling.

## Results

**Figure 1:**
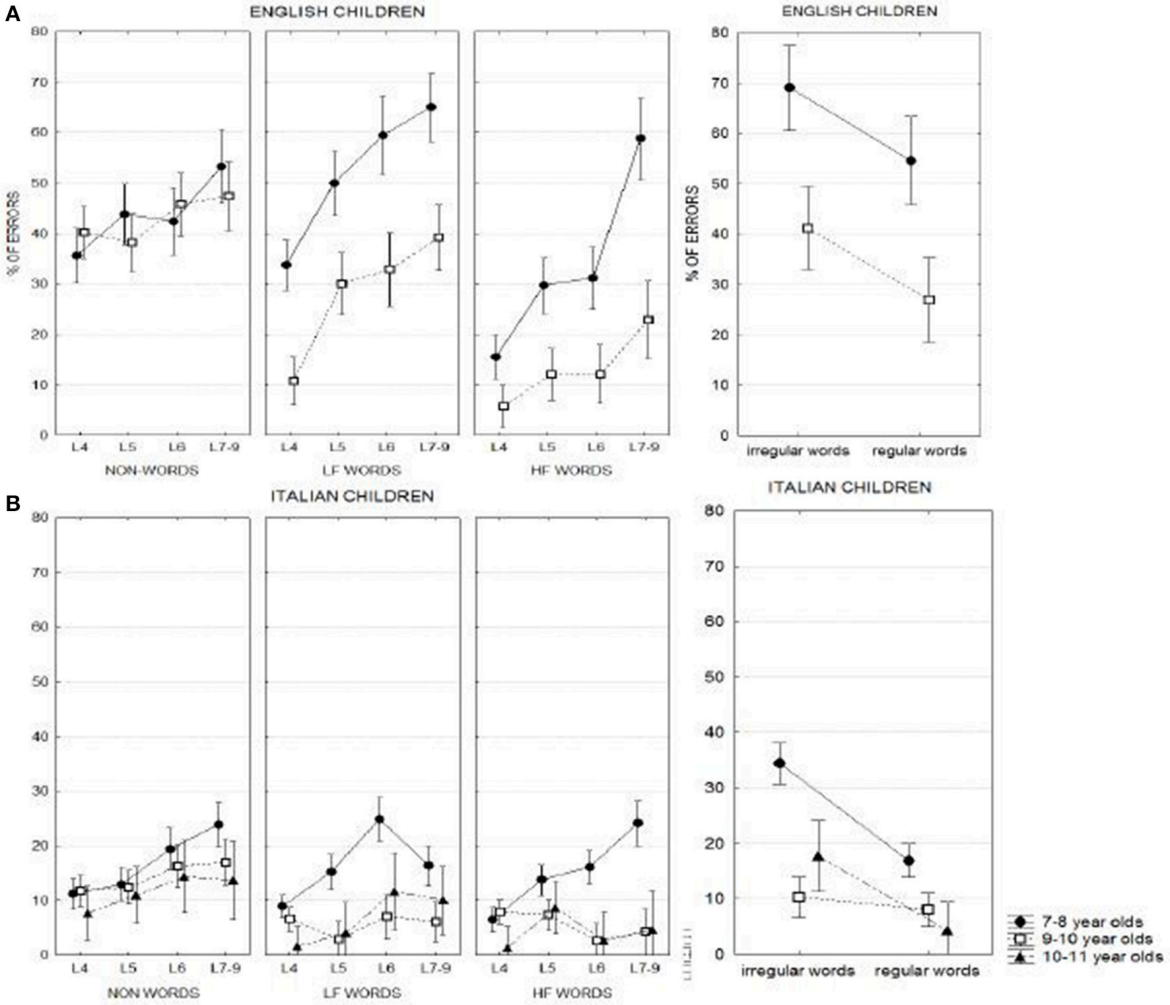
**Mean percentage of errors in spelling different types of stimuli as a function of age in English (upper part) and Italian (bottom part) children. (A)** Shows the children performance in spelling non-words, low frequency words and high frequency words; **(B)** represents the performance in spelling regular and irregular words. Lf, low frequency; hf, high frequency. L4, L5, L6, and L7–9 indicate respectively 4-, 5-, 6-, and 7–9- letters stimuli.

Main conditions are illustrated in Figure 1 separately for the English and Italian samples as a function of age. An inspection of these plots allows for a number of general observations:

- English children generally performed worse than Italian children across all conditions; performance of Italian (but not English) children was near ceiling for high frequency words by the age of 9–10 years and for low frequency words by the age of 10–11 years;
- Improvement in performance with age was marked for words but not (or much less) for non-words;
- Frequency, lexicality and regularity effects were all present and clear although they varied across ages and language groups; length modulated these effects in interaction with age.

Table 1 reports the results of the ANOVAs comparing children on the basis of age (“Chronological age comparison”), i.e., younger children (7–8 year old) and older children (9–10 year old) of both languages. In the section “Years of schooling comparison” (Table 2), English and Italian children were matched on the basis of the number of years of schooling, i.e., 5th grade Italian (10–11 year old) and English (9–10 year old) children were compared.

**Table 1:** **Results of the ANOVAs on spelling data for the chronological-age comparisons**.

**Effect**	***Df***	***F***	***P***	***_p_*η**^2^	**Reading *_p_* η^2^**
**1ST ANALYSIS: FREQUENCY EFFECT**					
Language	1, 251	84.46	0.000^***^	0.25	−0.01
Age	1, 251	45.88	0.000^***^	0.15	0.13
Frequency	1, 251	284.7	0.000^***^	0.53	0.41
Length	3, 753	157.89	0.000^***^	0.39	0.14
Language by age	1, 251	4.67	0.032^*^	0.02	0.00
Frequency by language	1, 251	242.57	0.000^***^	0.49	0.02
Frequency by age	1, 251	3.38	0.067	−0.01	0.19
Frequency by language by age	1, 251	0.51	0.447	0.00	0.04
Length by language	3, 753	76.05	0.000^***^	0.23	0.04
Length by age	3, 753	27.95	0.000^***^	0.10	0.06
Length by language by age	3, 753	4.55	0.004^**^	0.02	0.03
Frequency by length	3, 753	23.33	0.000^***^	0.09	0.02
Frequency by length by language	3, 753	4.46	0.004^**^	0.02	0.07
Frequency by length by age	3, 753	13.94	0.000^***^	0.05	0.02
Frequency by length by age by language	3, 753	1.31	0.269	−0.01	0.01
**2ND ANALYSIS: LEXICALITY EFFECT**					
Language	1, 245	10.66	0.000^***^	0.29	−0.01
Age	1, 245	16.95	0.000^***^	0.06	0.15
Lexicality	1, 245	221.84	0.000^***^	0.48	0.59
Length	3, 735	93.82	0.000^***^	0.28	0.16
Language by age	1, 245	1.31	0.253	−0.01	−0.01
Lexicality by language	1, 245	74.26	0.000^***^	0.23	−0.01
Lexicality by age	1, 245	63.12	0.000^***^	0.20	0.14
Lexicality by language by age	1, 245	14.28	0.000^***^	0.06	0.00
Length by language	3, 735	19.56	0.000^***^	0.07	0.11
Length by age	3, 735	28.18	0.000^***^	0.10	0.04
Length by language by age	3, 735	2.53	0.056	0.01	0.04
Lexicality by length	3, 735	13.9	0.000^***^	0.05	−0.01
Lexicality by length by language	3, 735	17.21	0.000^***^	0.07	0.03
Lexicality by length by age	3, 735	11.33	0.000^***^	0.04	0.01
Lexicality by length by age by language	3, 735	1.43	0.232	−0.01	0.01
**3RD ANALYSIS: REGULARITY EFFECT**					
Language	1, 251	133.8	0.000^***^	0.35	
Age	1, 251	7.33	0.000^***^	0.22	
Regularity	1, 251	203.39	0.000^***^	0.45	
Language by age	1, 251	4.51	0.035^*^	0.02	
Regularity by language	1, 251	6.78	0.01^**^	0.03	
Regularity by age	1, 251	2.86	0.000^***^	0.08	
Regularity by language by age	1, 251	19.67	0.000^***^	0.07	

* p < 0.05;

** p < 0.01;

*** p < 0.001.

Effect size is the partial eta squared (_p_η ^*2*^; in brackets values relative to main effects or interactions insignificant with < 0.05). For comparison, _p_η^*2*^ on similar analyses on reading performance are reported (when available). Following Cohen (1988, p. 283), 0.0099 is a reference point for a small effect, 0.0588 for a medium effect and 0.1379 for a large effect.

**Table 2:** **Results of the three ANOVAs on spelling data for the years of school comparison**.

**Effect**	***Df***	***F***	***p***	***_p_* η^2^**	**Reading *_p_* η^2^**
**1ST ANALYSIS: FREQUENCY EFFECT**					
Language	1, 96	21.5	0.000^***^	0.18	−0.03
Frequency	1, 96	108.08	0.000^***^	0.59	0.25
Length	3, 288	45.54	0.000^***^	0.38	0.04
Frequency by language	1, 96	55.14	0.000^***^	0.42	−0.01
Length by language	3, 288	17.29	0.000^***^	0.17	−0.08
Frequency by length	3, 288	11.73	0.000^***^	0.14	0.04
Frequency by length by language	3, 288	8.49	0.000^***^	0.09	0.06
**2ND ANALYSIS: LEXICALITY EFFECT**					
Language	1, 86	28.84	0.000^***^	0.30	−0.02
Lexicality	1, 86	63.24	0.000^***^	0.64	0.52
Length	3, 258	18.31	0.000^***^	0.17	0.10
Lexicality by language	1, 86	18.59	0.000^***^	0.39	0.00
Length by language	3, 258	9.16	0.000^***^	0.08	−0.07
Lexicality by length	3, 258	4.75	0.003^**^	0.07	0.03
Lexicality by length by language	3, 258	2.74	0.044^*^	0.04	0.03
**3RD ANALYSIS: REGULARITY EFFECT**					
Language	1, 96	26.26	0.000^***^	0.21	
Regularity	1, 96	132.25	0.000^***^	0.60	
Regularity by language	1, 96	0.44	0.507	0.00	

* p < 0.05;

** p < 0.01;

*** p < 0.001.

Effect size is the partial eta squared (_p_η ^*2*^; in brackets values relative to main effects or interactions insignificant with < 0.05). For comparison, _p_η ^*2*^ on similar analyses on reading performance are reported (when available). Following Cohen (1988, p. 283), 0.0099 is a reference point for a small effect, 0.0588 for a medium effect and 0.1379 for a large effect.

### Frequency effect: chronological age comparison

As presented in Table 1, the ANOVA showed the significant and large size main effects of *language* [*F*_(1, 251)_ = 84.46, *p* < 0.0001], *age* [*F*_(1, 251)_ = 45.88, *p* < 0.0001], *frequency* [*F*_(1, 251)_ = 284.70, *p* < 0.0001], and *length* [*F*_(3, 753)_ = 157.89, *p* < 0.0001], indicating lower percentage of errors for Italian (*M* = 10.7%, *SD* = 16.1) than English children (*M* = 30.8%, *SD* = 16.2), older (*M* = 13.3%; *SD* = 17.4) than younger children (*M* = 28.2%, *SD* = 17.4), high (*M* = 16.5%, *SD* = 17.1) than low frequency words (*M* = 25.0%, *SD* = 18.8) and shorter than longer words (from *M* = 11.6%, *SD* = 13.7 to *M* = 29.3%, *SD* = 22.5- passing from 4- to 7–9- letter words).

**Figure 2:**
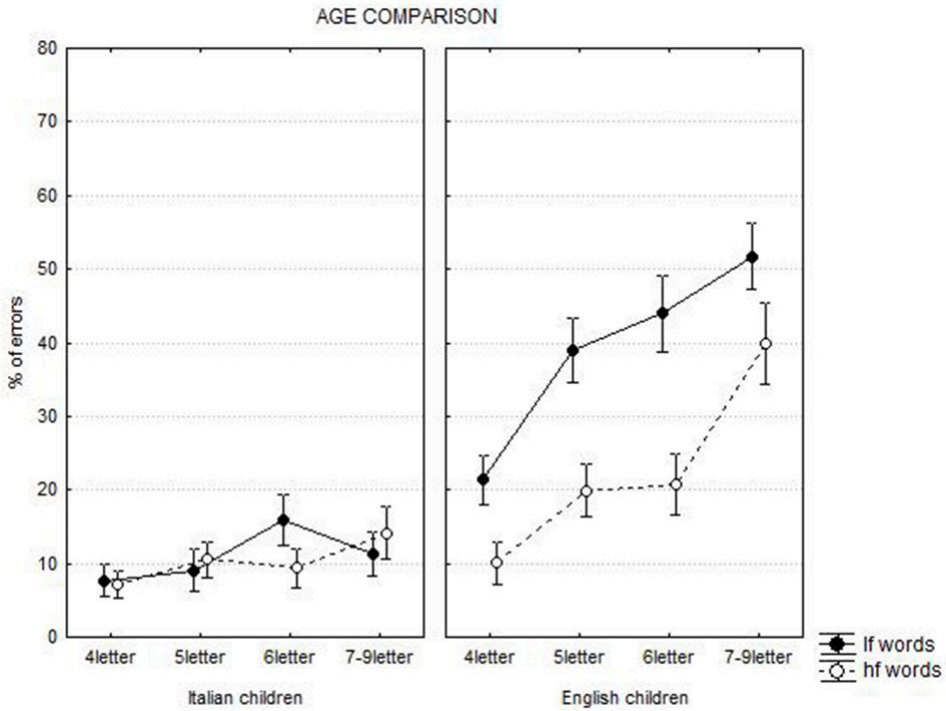
**Chronological age comparison: frequency by length by language interaction**. Lf, low frequency words; hf, high frequency words.

The *frequency* by *length* by *language* interaction [*F*_(3, 753)_ = 4.46, *p* < 0.01] is presented in Figure 2. The frequency effect was larger (and similar for each words length) for English (mean diff. = 16%, at least *p* < 0.0001) than Italian children. English children made many errors in spelling low frequency words and the number of errors was progressively higher with increasing word length (mean increase per letter = 10%, at least *p* < 0.001). Also in spelling high frequency words English children made more errors with longer words (mean increase per letter = 10%, at least *p* < 0.0001), except in the case of 5- and 6-letter words. In Italian children, the frequency effect was negligible at all lengths except for 6-letter stimuli.

The *frequency* by *length* by *age* interaction [*F*_(3, 753)_ = 13.94, *p* < 0.0001] indicated that, for low frequency words, there was a lower performance in spelling longer words in both younger and older children; in the case of high frequency words, this pattern was clear only among younger children.

The *language* by *age* interaction was significant [*F*_(1, 251)_ = 4.67, *p* < 0.05], indicating a greater increase in performance with age among English than Italian children (11.6 vs. 10.1%, respectively). *Language* and *age* interacted also with *length* [*F*_(3, 753)_ = 4.55, *p* < 0.01]. In English, improvement in performance with age was similar at each length (mean diff. = 20%, at least *p* < 0.0001); in Italian, younger children had a performance similar to that of older children in the case of short words and the difference between older and younger children emerged only in the case of longer words (mean diff. = 13%, at least *p* < 0.0001). English children had always a worse performance than Italian children (mean diff. between groups = 22.9%; at least *p* < 0.0001), with the exception of very short words (4 letters) that were spelled with similar accuracy by older children of both languages (diff. between groups = 1.4%, ns). A large increase of the number of errors as a function of word length was present among younger and older English children (mean increase per letter = 12 and 8%, respectively; with the exception of 5- and 6- letter words that were spelt with similar accuracy). In Italian children, the effect of length was present for younger children, even though to a smaller extent than in younger English children (mean increase per letter = 6%, at least *p* < 0.0001, with the exception of 6- and 7-9-letter words that were spelt with similar levels of accuracy). The length effect was not displayed by older children (mean increase per letter = 0%).

### Frequency effect: years of schooling comparison

As presented in Table 2, the ANOVA showed the main effects of *language* [*F*_(1, 96)_ = 21.50, *p* < 0.0001], *frequency* [*F*_(1, 96)_ = 108.08, *p* < 0.0001], and *length* [*F*_(3, 288)_ = 45.54, *p* < 0.0001], indicating lower percentage of errors in Italian (*M* = 5.6%, *SD* = 16.9) than in English children (*M* = 22.7%, *SD* = 16.9), with high (*M* = 9.8%, *SD* = 17.5) than low frequency words (*M* = 18.5%, *SD* = 19.9) and with shorter than longer words (progressively from *M* = 5.6%, *SD* = 13.7 to *M* = 21.0%, *SD* = 25.2, passing from 4- to 7–9-letter words).

All first grade interactions were significant, as well as the *frequency* by *length* by *language* interaction [*F*_(3, 288)_ = 8.49, *p* < 0.0001]. The frequency effect was larger in English than Italian children (*p* < 0.0001) and it was present for all word lengths (mean diff. = 15%, at least *p* < 0.01). For Italian children it was small (mean diff. = 2.5%) and significant only for 6-letter words (*p* < 0.01). In the case of low frequency words, for English children the number of error increased as a function of word length (mean increase per letter = 9.9%, at least *p* < 0.0001); increase per letter was small (2.8%) and not significant in Italian children. For high frequency words, only English children showed more errors with longer words (mean increase per letter = 6.9%, at least *p* < 0.05); for Italian children, errors were below 10% at each length considered. Note that with high frequency words (especially for shorter words), older English children were able to compensate their spelling difficulty and obtained similar performance to that of 5th grade Italian children.

### Frequency effect: brief summary of results

The English children displayed higher percentages of errors than Italian children in both the age and school comparisons. A greater increase in performance with age emerged in English than Italian children.

English children showed larger frequency effects than Italian ones. A large length effect was present among younger and older English children. In Italian children, the effect of length was present in younger children (although smaller compared to English children) and was not present in older children. This pattern was evident in both the age and school comparisons.

### Frequency effect: comparison with reading accuracy performance

For the sake of comparison, Tables 1, 2 report size effects (computed as partial eta squared) for spelling and reading performance on the same stimuli for the chronological age comparison and the years of schooling comparison, respectively. Notably, the size of the frequency effect is well above the reference point for a large effect (i.e., >0.1379) and similar in reading and spelling in the chronological age comparison; also in the years of schooling comparison the frequency effect is large in spelling and in reading though it is clearly smaller in this latter case. The most noticeable difference between the two tasks is the presence of a large size effect for language in the case of spelling but not reading for which no clear effect is indeed obtained. Furthermore, also interactions between language and frequency and between language and length indicate large size effects for spelling. No interaction with language reaches a large size effect in the case of reading; a medium size effect is present only in the case of the frequency by length by language interaction.

These comparisons indicate that, by the ages tested, general cross-linguistic differences in reading accuracy have largely resolved while they are still quite clear in the case of spelling.

### Lexicality effect: chronological age comparison

As presented in Table 1, the ANOVA showed the main effects of *language* [*F*_(1, 245)_ = 10.66, *p* < 0.0001], *age* [*F*_(1, 245)_ = 16.95, *p* < 0.0001], *lexicality* [*F*_(1, 245)_ = 221.84, *p* < 0.0001], and *length* [*F*_(3, 735)_ = 93.82, *p* < 0.0001], indicating fewer errors for Italian (*M* = 13.0%, *SD* = 14.6) than English children (*M* = 33.4%, *SD* = 14.6), for older (*M* = 19.0%; *SD* = 15.9) than younger children (*M* = 27.4%, *SD* = 16.4), for words (*M* = 16.9%; *SD* = 15.8) than non-words (*M* = 29.5%, *SD* = 15.8), and for shorter than longer stimuli (from *M* = 16.8%, *SD* = 13.4 to *M* = 31.4%, *SD* = 22.9) passing from 4- to 7–9- letter stimuli). With the exception of the *language* by *age* and the *language* by *age* by *length* by *lexicality*, all interactions were significant.

**Figure 3:**
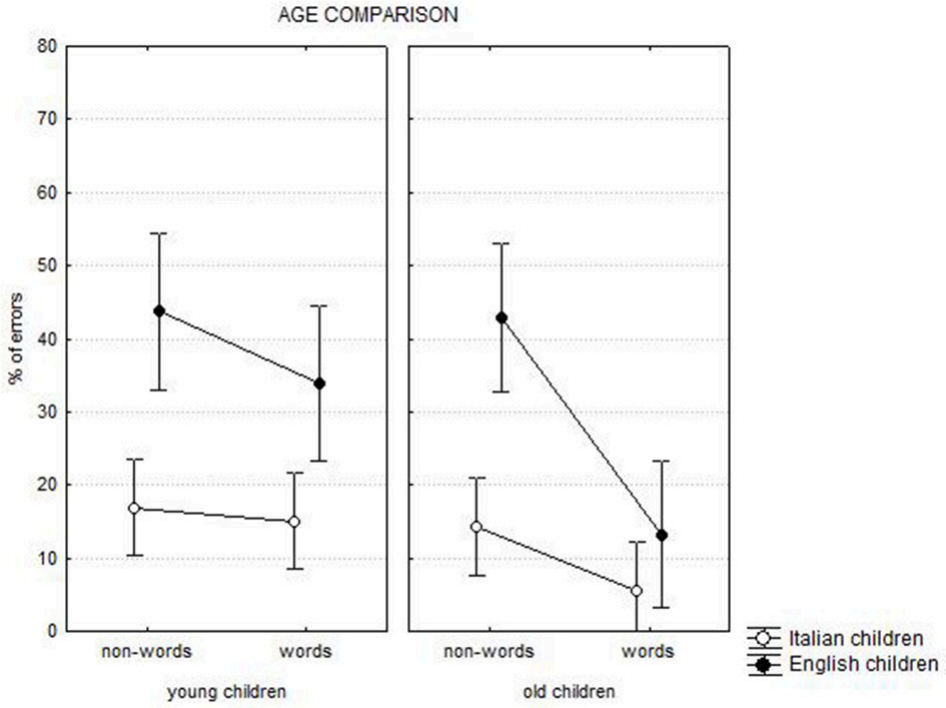
**Chronological age comparison: language by age by lexicality interaction**.

The *language* by *age* by *lexicality* interaction [*F*_(1, 245)_ = 14.28, *p* < 0.0001] is presented in Figure 3. The lexicality effect was present for all groups (at least *p* < 0.0001) with the exception of younger Italian children. English children showed larger lexicality effects than Italian children in younger (diff. = 9.9 vs. 1.8%, respectively, at least *p* < 0.0001) and especially in older children (diff. = 8.8 vs. 29.7%, respectively, at least *p* < 0.0001). Larger cross-linguistic differences were present in spelling non-words compared to words (diff. = 27.7 vs. 13.2%, respectively, at least *p* < 0.0001), with more errors among English than Italian children. Moreover, the spelling performance improved with age to a larger extent in spelling words than in spelling non-words in Italian (diff. = 2.5 and 9.6% for words and non-words respectively, *p* < 0.0001), and especially in English children (diff. = 0.9 vs. 20.0% for words and non-words respectively, *p* < 0.0001).

*Language* and *lexicality* also interacted with *length* [*F*_(3, 735)_ = 17.21, *p* < 0.0001]: at each length, except for 7–9-letter stimuli, the lexicality effect was larger (at least *p* < 0.0001) in English (mean = 23%) than in Italian (5%) children. In the case of non-words, spelling errors increased progressively as a function of the number of letters to be spelled, an effect similar in both languages. Then, cross-linguistic differences in spelling non-words were large but not modulated by length. In the case of words, length modulated the spelling performance of English but not of Italian children (for whom the percentage of errors were quite low). Note that the percentage of errors in spelling words decreased substantially for English children, especially for shorter stimuli. This produced smaller cross-linguistic differences compared to non-words, especially for shorter stimuli.

The *language* by *length* by *age* interaction approached significance [*F*_(3, 735)_ = 2.53, *p* = 0.056]: there was an effect of length in younger children of both languages (mean of increase for letter = 10.1 and 5.1% for English and Italian, respectively); among older children, the effect of length was present for English, but not Italian, children (4.0 and 0.3%, respectively).

The *age* by *length* by *lexicality* interaction [*F*_(3, 735)_ = 11.33, *p* < 0.0001] indicated that, among younger children, the advantage in spelling words with respect to non-words was progressively smaller as length increased; length did not modulate the lexicality effect among older children (for whom the lexicality effect was generally larger; diff. = 19 vs. 6%, respectively). Performance improved with age in the case of words (by about 15.1%) but not in the case of non-words (1.7%).

### Lexicality effect: years of schooling comparison

As presented in Table 2, the ANOVA showed the main effects of *language* [*F*_(1, 86)_ = 28.84, *p* < 0.0001], *lexicality* [*F*_(1, 86)_ = 63.24, *p* < 0.0001], and *length* [*F*_(3, 258)_ = 18.31, *p* < 0.0001], indicating fewer errors in Italian (*M* = 8.0%, *SD* = 16.2) than in English children (*M* = 27.6%, *SD* = 16.2), with words (*M* = 9.8%, *SD* = 17.3) than with non-words (*M* = 25.8%, *SD* = 17.3), and with shorter compared to longer stimuli (from *M* = 13.3%, *SD* = 14.6 to 22.7%, *SD* = 22.8, passing from 4- to 7–9-letter stimuli).

All first grade interactions were significant, as well as the *lexicality* by *length* by *language* interaction [*F*_(3, 258)_ = 2.74, *p* < 0.05]. The lexicality effect was greater among English than Italian children (*p* < 0.0001), being present for all lengths (mean diff. = 24.7%, at least *p* < 0.0001); it was smaller for Italian children (mean diff. = 7.3%) and significant only for the 6- and 7–9-letter stimuli (at least *p* < 0.05). Number of errors increased as a function of word length in English for both words and non-words (mean of increase for letter = 3.1 and 6.4%, respectively); in Italian, the effect of length was apparent only in the case of non-words (mean of increase for letter = 2%).

### Lexicality effect: brief summary of results

A language effect, with lower performance in English as compared to Italian children, was evident in both types of analysis. English children, especially older ones, showed larger lexicality effects than Italian children. This cross-linguistic difference was evident also when children were compared in terms of years of schooling. The lexicality effect was present for all groups, except for younger Italian children.

Cross-linguistic differences in spelling non-words were large but length effects were similar in the two languages. In the case of words, length modulated the spelling performance of English, but not Italian, children (for whom error rates were quite low). This pattern was confirmed in the analysis comparing English and Italian children in terms of years of schooling.

### Lexicality effect: comparison with reading accuracy performance

For comparison, Tables 1, 2 report size effects for spelling and reading performance on the same stimuli for the chronological age comparison and the years of schooling comparison, respectively. Notably, the lexicality effect is very large and similar in reading and spelling in both chronological and years of schooling comparisons. Language has a large effect size (both as a main effect and in interaction with lexicality) in the case of spelling; furthermore, a large size effect marks the interaction between language and lexicality. The direct effect of language is negligible in the case of reading accuracy where only a medium size interaction with length is detected.

### Regularity effect: chronological age comparison

As presented in Table 1, the ANOVA showed the main effects of *language* [*F*_(1, 251)_ = 133.80, *p* < 0.0001], *age* [*F*_(1, 251)_ = 7.33, *p* < 0.0001], and *regularity* [*F*_(1, 251)_ = 203.39, *p* < 0.0001], indicating lower percentage of errors for Italian (*M* = 17.5%; *SD* = 19.4) than English children (*M* = 48.0%, *SD* = 20.6), older (*M* = 21.7%, *SD* = 20.8) than younger children (*M* = 43.8%; *SD* = 20.8), and regular (*M* = 26.7%; *SD* = 21.2) with respect to irregular words (*M* = 38.8%; *SD* = 21.2).

**Figure 4:**
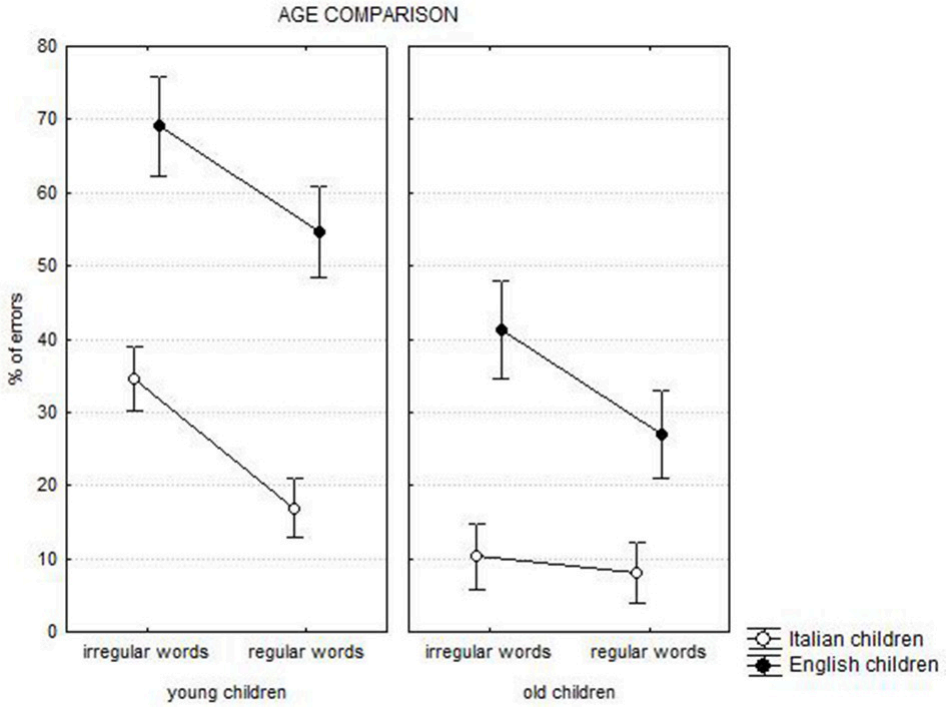
**Chronological age comparison: regularity by language by age interaction**.

The ANOVA also showed the significance of all first grade interactions, as well as the *regularity* by *language* by *age* interaction [*F*_(1, 251)_ = 19.67, *p* < 0.0001; see Figure 4]. An inspection of the figure indicates many more errors among English than Italian children. A regularity effect was present for both young and old English children (effect = 14.5 and 14.3%, respectively, at least *p* < 0.0001), but only in younger (effect = 17.6%, *p* < 0.0001) and not in older (2.3%, ns) Italian children. Despite English children had worse performance than the Italian ones, the regularity effect was similar for the two groups in the case of younger children. By contrast, among older children the regularity effect was larger in English than in Italian children (*p* < 0.0001).

### Regularity effect: years of schooling comparison

As presented in Table 2, the analysis highlighted the main effects of *language* [*F*_(1, 96)_ = 26.26, *p* < 0.0001] and *regularity* [*F*_(1, 96)_ = 132.25, *p* < 0.0001], indicating lower percentages of errors in Italian (*M* = 11.1%, *SD* = 21.9) than in English children (*M* = 35.7%; *SD* = 22.0), and in spelling regular (*M* = 16.3%, *SD* = 24.1) than irregular words (*M* = 30.5%, *SD* = 25.1).

The *regularity by language* interaction was not significant [*F*_(1, 96)_ = 0.44, *p* > 0.05]: word regularity modulated spelling performance in the same way in both orthographies among 5th grade children. In fact, the disadvantage in spelling irregular as compared to regular words was similar in Italian and English children (diff. = 13.4 and 15.1%, respectively); furthermore, the disadvantage of English children with respect to Italian children was similar in regular (diff. = 23.9%) and irregular words (diff. = 25.5%).

### Regularity effect: brief summary of results

A language effect was present in both the age and school comparisons, with more errors among English than Italian children.

The regularity effect was similar for the two languages for younger children, while it was larger in English than in Italian children for older children (for whom the low percentage of errors made the regularity effect not detectable). In the years of schooling comparison, a main effect of language was still evident (with lower performance in English compared to Italian children), but the interaction with regularity was not present: regularity modulated spelling performance in the same way in English and Italian 5th grade children.

## Discussion

Although there are several reasons to assume that orthographic consistency modulates spelling acquisition to a greater extent than reading, only few cross-linguistic studies have examined spelling acquisition in relation to the consistency of the orthography. In the present study, we examined spelling acquisition in Italian and English children as a function of age and found greater accuracy in spelling among Italian than English children. This effect was clear in the case of the samples matched for chronological age and it held true in the case of samples matched for the number of years of schooling. Therefore, cross-linguistic differences in spelling performance cannot be easily accounted for by differences in the timing of formal instruction in the two countries. Rather, the lower accuracy of English children indicates the greater spelling difficulty of an inconsistent orthography, both for the larger number of irregular words to be learned and the difficulty in acquiring the rules of conversion between phonemes and graphemes (and *vice versa*). The general pattern of the present results appears consistent with previous studies highlighting higher accuracy in spelling among shallow orthographies (Bruck et al., 1996, Unpublished raw data; Bruck et al., 1998).

However, some notes of caution seem in order when interpreting the obtained cross-linguistic differences. First, one should keep in mind that there are intrinsic limitations in fully matching the linguistic characteristics of the stimuli used in the two languages. Thus, whereas in English irregular words are inconsistent in their sound-letter correspondence, “irregular” words in Italian may be better characterized as words with a spelling unpredictable according to sound-letter knowledge (because they have more than one phonologically plausible orthographic solutions). Therefore, while in both languages the correct spelling of irregular words requires the child to know the given word, the number of spelling solutions is appreciably lower in Italian than those of the corresponding English irregular words. Accordingly, the chance level of spelling a word correctly is lower for English than Italian spellers. At any rate, as discussed below, crosslinguistic effects emerged in the way spelling was modulated by psycholinguistic variables, indicating that differences are qualitative and go beyond the greater task difficulty in spelling English words.

Second, some limitations in the characteristics of actual samples examined should be noted. We have chosen to consider Italian and English schools with similar teaching methods; furthermore, all belonged to middle socio-economic backgrounds. However, in the sample selection it was not possible to control for other variables that might influence spelling acquisition, such as teacher effectiveness, experience and competence, as well as the quantity of exposure to print at home or the socio-economic background (for a systematic review on variables affecting literacy see Marinelli et al., 2012). Finally, a difference in sample size (larger in Italian with respect to English participants) was present. All these differences in the composition of our samples should be kept in mind when interpreting the pattern of findings of the present study.

As expected, spelling was generally more effective than reading in producing cross-linguistic differences. In reading the language effect was quite small in absolute terms and not significant across conditions, even if it was detectable with specific linguistic stimuli (Marinelli et al., submitted); by contrast, in the case of spelling the language effect was much larger and always significant across conditions. This pattern might be explained by the greater task demand in spelling, as well as the need of fully specified representations for spelling compared to reading (e.g., Perfetti, 1992). In fact, partial lexical representations may provide enough information for correct naming, but they are not detailed enough for accurate spelling (Tainturier and Rapp, 2001; see also Angelelli et al., 2010). An alternative hypothesis is that larger cross-linguistic differences emerge in spelling compared to reading because English is even more irregular in spelling compared to reading (Kessler and Treiman, 2001). However, it is important to note that also Italian is more inconsistent in spelling compared to reading. In any case, higher inconsistency of English compared to Italian orthography was present also in spelling.

A critical aim of the present study was to examine how children of both languages were influenced by the psycholinguistic characteristics of the stimuli to be spelt. In particular, the effects of frequency, lexicality, length, and regularity were examined.

Italian children had a near ceiling performance for both high- and low-frequency words; greater difficulty in spelling non-words compared to words was detectable only among older children while younger children had similar performance with the two types of stimuli. It seems that Italian children are advantaged when they can use both the lexical and sub-lexical procedures, as in the case of words (compared to non-words). However, it is important to note that in spelling non-words the extremely high consistency in the phoneme-to-grapheme mapping allows obtaining relatively high levels of accuracy throughout the sub-lexical procedure. With increased age and spelling experience, the performance on words improved compared to that on non-words and the spelling accuracy was not modulated yet by length. Moreover, with age Italian children did not show the regularity effect any longer. Probably, they acquired the lexical representations of irregular words and then were not disadvantaged in spelling these stimuli compared to real words. In fact, a consistent phoneme-grapheme mapping not only implies that irregular words are only a few in the language, but also guarantees an easier acquisition of phoneme-to-grapheme mappings.

English children had lower levels of accuracy in spelling overall. Their performance was clearly modulated by length; further, large frequency and lexicality effects, as well as the regularity effect, were present in both younger and older children. These findings indicate that also 5th grade children take advantage in spelling words for which both procedures can be used but their performance remains below the mean of Italian children even in this case. Also, sub-lexical spelling was quite difficult for English children. English inconsistency may produce this low accuracy in spelling for two reasons: (i) the inconsistence of the phoneme-to-grapheme mapping made more difficult to acquire the conversion rules; (ii) the lexical spelling is more demanding due higher number of orthographic representations to be learnt. Furthermore, difficulties in sub-lexical conversion may indirectly hinder the acquisition of orthographic knowledge. According to Ehri (1998), as in the case of phonological reading, also phonological spelling has a self-teaching function in the acquisition of orthographic knowledge, because it requires the focus on orthographic details and sub-lexical print-to-sound relationships. In fact, as suggested by the “self-teaching” hypothesis (Jorm and Share, 1983; Share, 1995, 2008; Shahar-Yames and Share, 2008), phonological decoding not only allows for the identification of new words but also for establishing detailed word-specific orthographic representations.

As stated by the *orthographic depth hypothesis* (Katz and Frost, 1992), readers adapt their reliance on one or another of the procedures depending on the orthographic properties of the language. In shallow orthographies, the phonological route would be preferred, while in deep orthographies readers would be encouraged to use the direct lexical/orthographic route because grapheme-phoneme correspondences are often equivocal (e.g., Frost, 1994). In fact, while Italian is very consistent at a small grain size, the English orthography is very inconsistent at the level of units of a small size, while it is more regular at larger grain sizes (Treiman et al., 1995). This characteristic of the English orthography may induce children to learn the correspondences of bigger orthographic units, such as patterns of letters, rhymes, syllables and whole words (Ziegler and Goswami, 2005). This result has been reported in cross-linguistic studies on reading that indicate the use of larger units of analysis in more inconsistent orthographies compared to more consistent ones (Ziegler et al., 2003). Similarly, in the reading part of the present study, we evidenced a tendency of English children to use larger grain sizes than Italian children (see Marinelli et al., submitted, for an in-depth description of these results).

Note that, according to recent models of spelling, large grain size units also play a role in spelling (Perry and Ziegler, 2004): individuals not only spell by retrieving whole words, but are also sensitive to the frequency of occurrence of large size sub-syllabic sound-spelling relationships beyond phoneme-grapheme correspondences. This effect has been explored in English and only recently in more consistent orthographies (Angelelli et al., 2014). This suggests that “*people's spelling needs to be understood in terms of both the relationships between sound and spelling at various grain sizes and purely orthographic frequency at and below the whole word level* (Perry and Ziegler, 2004; p. 348).” Then, it is possible that orthographic consistency modulates also the use of large-size sound-spelling relationships. Studies need to be carried out in order to explore this aspect more thoroughly.

In conclusion, even second grade Italian children had little difficulty in spelling and older fourth and fifth grade children showed to have acquired the orthographic representation of irregular words. Probably, the high consistency of Italian allowed obtaining high accuracy with the phoneme-grapheme conversion rules and, then, to early acquire the lexical representation of the few words with an unpredictable transcription. By contrast, English inconsistency resulted in a much lower performance at each age tested. Moreover, despite also English older children took advantage in spelling words for which both lexical and sub-lexical procedures might be used, their performance was at any rate lower with respect to that of Italian children. English children also had difficulty in the phoneme-grapheme conversion skills, and this may (in turn) have contributed to a difficulty in acquiring irregular words (Share, 2008) that represent a large proportion of English words. Cross-linguistic differences in spelling accuracy proved to be more persistent than the corresponding ones in reading accuracy; by the age tested, language did not affect reading accuracy appreciably in keeping with the idea that spelling (but not reading) requires fully specified lexical representations.

### Conflict of interest statement

The authors declare that the research was conducted in the absence of any commercial or financial relationships that could be construed as a potential conflict of interest.

## Footnotes

^1^However, it is important to keep in mind that the percentage of spelling errors increases as a function of length also for probabilistic reasons (because of the increase in the probability to make a misspelling) or for the larger involvement of other abilities, such as phonological short term memory or working memory.

^2^According to this criterion, four children with a performance worse than 1.5 standard deviations in the Raven's Colored Progressive Matrices were not included in the sample.

^3^Note that, in England, children are admitted to first grade if they have reached the appropriate age by August-September, while, in Italy, this limit is delayed until December. This difference is presumably responsible for the difference in the ages of Italian and English samples.

^4^Note that both Italian and English word frequencies are calculated over 1,000,000 occurrences.

^5^Except for a word with non-dominant stress that accidentaly has been included in the lists.
