# Supplementary material for: Spelling Acquisition in English and Italian: A Cross-Linguistic Study
Source: Front Psychol. 2015 Dec 8;6:1843. doi: 10.3389/fpsyg.2015.01843 (PMC4672065; doi:10.3389/fpsyg.2015.01843)
Supplement: Supplementary file 1 [file DataSheet1.DOCX]

## Appendix 1.

Italian and English lists used to assess the effects of frequency, lexicality and length (N = 120 stimuli).

ITALIAN HIGH FREQUENCY WORDS:

**4- letters:** zona, mago, erba, seta, lato, base, foto, male, gara, arte;

**5- letters:** vasca, barba, sedia, ansia, bomba, latte, sonno, ombra, festa, fuoco;

**6- letters:** matita, sabbia, nipote, moneta, affare, fretta, denaro, sangue, estate, gruppo;

**7-9- letters:** femmina, negozio, inverno, soffitto, ghiaccio, finestra, silenzio, ospedale, famiglia, bicchiere.

ITALIAN LOW FREQUENCY WORDS:

**4- letters:** rapa, bava, sega, elmo, orma, lode, zelo, boia, lino, nuca;

**5- letters:** gobba, manzo, garza, muffa, sorso, rospo, sacca, astro, rissa, aglio;

**6- letters:** fibbia, dogana, seppia, biscia, ovatta, laccio, cipria, mancia, avorio, flotta;

**7-9- letters:** lampone, bacheca, diluvio, cipresso, aragosta, lanterna, merluzzo, cisterna, arsenale, monastero.

ITALIAN NON-WORDS:

**4- letters:** zila, muci, esfi, siba, libo, bipo, fipo, mafe, gomi, urse;

**5- letters:** nisca, bilfa, sipio, arsia, bumbo, libbe, sinno, ostra, fisti, fuago;

**6- letters:** matoba, sibbio, nulote, minefa, affime, fietta, deparo, sancio, espele, chippo;

**7-9- letters:** reffina, nagopio, isterno, soffatto, ghioggia, fibestre, sipinzio, ostedane, firiglia, bucchiole.

ENGLISH HIGH FREQUENCY WORDS:

**4- letters:** card, club, farm, game, lady, milk, neck, roof, sand, soil;

**5- letters:** clock, cloth, flame, paper, party, plane, pound, radio, storm, table;

**6- letters:** border, branch, family, jungle, letter, number, office, silver, volume, winter;

**7-9- letters:** article, blanket, example, husband, morning, opinion, partner, distance, hospital, direction.

ENGLISH LOW FREQUENCY WORDS:

**4- letters:** ramp, mink, plum, scar, cork, brim, cube, crib, wink, sage;

**5- letters:** tulip, moose, latch, pedal, spice, trash, spade, cider, gravy, glove;

**6- letters:** goblet, cavern, bandit, buckle, pimple, stripe, pickle, hurdle, beetle, napkin;

**7-9- letters:** emerald, hamster, lobster, inferno, scooter, scorpion, cucumber, pendulum, ornament, dandelion.

ENGLISH NON-WORDS:

**4- letters:** sarn, klib, nard, zale, maly, gilm, leck, zoof, dant, cois;

**5- letters:** glock, ploth, glame, naver ,narvy, flape, cound, panio, stoln, saple;

**6- letters:** vorner, tranch, daminy, jumple, retter, zunder, ollice, dilber, colune, sinter;

**7-9- letters:** andicle, clandet, exandle, huscant, horping, omidion, dartyer, bisbance, gosbital, tinection.

## Appendix 2

List of words (N = 60) with regular and irregular spelling for Italian and English.

IRREGULAR ENGLISH WORDS:

Horror, cotton, tennis, mirror, college, school, sapphire, country, friend, village, marriage, machine, island, breakfast, valley, mountain, stomach, concrete, ceiling, journal, jersey, antique, submarine, monkey, shield, leopard, trolley, spinach, jockey, pigeon.

REGULAR ENGLISH WORDS:

Father, ground, evening, president, market, capital, minister, paradise, monument, bronze, degree, station, student, shoulder, forest, poverty, cabinet, artist, victim, planet, platform, hunger, nursery, temple, carpet, basket, referee, plunge, timber, alcohol.

IRREGULAR ITALIAN WORDS:

Acqua, cuoio, cuoco, cuore, innocuo, scuola, cielo, specie, superficie, igiene, hanno, discepolo, mascella, ruscello, sceriffo, squalo, squama, sequenza, aquila, liquore, obliquo, quota, quotidiano, anno, ancella, concerto, macello, sergente, coscienza, scienza.

REGULAR ITALIAN WORDS:

Nuovo, battesimo, bussola, vescovo, candela, carriera, cantiere, canzone, capriccio, carota, categoria, cimitero, cristallo, cronaca, farina, epoca, fieno, guancia, guerra, segreto, luogo, fanno, mestiere, coma, piombo, preda, religione, scorpione, statua, vita.
